# Seasonal changes in N-cycling functional genes in sediments and their influencing factors in a typical eutrophic shallow lake, China

**DOI:** 10.3389/fmicb.2024.1363775

**Published:** 2024-02-05

**Authors:** Ling Zhang, Junhong Bai, Yujia Zhai, Kegang Zhang, Yaqi Wang, Ruoxuan Tang, Rong Xiao, Milko A. Jorquera

**Affiliations:** ^1^School of Environment, Beijing Normal University, Beijing, China; ^2^School of Chemistry and Chemical Engineering, Qinghai Normal University, Xining, China; ^3^Department of Environmental Engineering and Science, North China Electric Power University, Baoding, China; ^4^College of Environment & Safety Engineering, Fuzhou University, Fuzhou, China; ^5^Laboratorio de Ecología Microbiana Aplicada (EMALAB), Departamento de Ciencias Químicas y Recursos Naturales, Universidad de La Frontera, Temuco, Chile

**Keywords:** sediments, N-cycling, functional genes, seasonal changes, shallow lake

## Abstract

N-cycling processes mediated by microorganisms are directly linked to the eutrophication of lakes and ecosystem health. Exploring the variation and influencing factors of N-cycling-related genes is of great significance for controlling the eutrophication of lakes. However, seasonal dynamics of genomic information encoding nitrogen (N) cycling in sediments of eutrophic lakes have not yet been clearly addressed. We collected sediments in the Baiyangdian (BYD) Lake in four seasons to explore the dynamic variation of N-cycling functional genes based on a shotgun metagenome sequencing approach and to reveal their key influencing factors. Our results showed that dissimilatory nitrate reduction (DNRA), assimilatory nitrate reduction (ANRA), and denitrification were the dominant N-cycling processes, and the abundance of *nirS* and *amoC* were higher than other functional genes by at least one order of magnitude. Functional genes, such as *nirS*, *nirK* and *amoC*, generally showed a consistent decreasing trend from the warming season (i.e., spring, summer, fall) to the cold season (i.e., winter). Furthermore, a significantly higher abundance of nitrification functional genes (e.g., *amoB, amoC and hao*) in spring and denitrification functional genes (e.g., *nirS, norC and nosZ*) in fall were observed. N-cycling processes in four seasons were influenced by different dominant environmental factors. Generally, dissolved organic carbon (DOC) or sediment organic matter (SOM), water temperature (T) and antibiotics (e.g., Norfloxacin and ofloxacin) were significantly correlated with N-cycling processes. The findings imply that sediment organic carbon and antibiotics may be potentially key factors influencing N-cycling processes in lake ecosystems, which will provide a reference for nitrogen management in eutrophic lakes.

## 1 Introduction

Nitrogen input caused by human activities can greatly affect the processes of the N-cycling of lake ecosystems, leading to the eutrophication of water bodies (Basu et al., 2022; Jiang et al., 2023). It has been proved microorganisms, especially N-cycling functional genes are the key driver of the nitrogen transformation processes in the lakes (Isobe and Ohte, 2014). Therefore, N-cycling functional genes have been given more and more concerns for nitrogen removal of the eutrophic lakes.

N-cycling plays an important role in maintaining the ecological balance of lakes (Isobe and Ohte, 2014). Nitrogen in lakes exists in the form of inorganic nitrogen and organic nitrogen, which is absorbed and assimilated by algae, macrophytes (Wu et al., 2021), benthic animals and other organisms (Wu Y. et al., 2022), and can be converted into biological organic nitrogen (Pajares et al., 2017). After these organisms die, they release a large amount of organic nitrogen and inorganic nitrogen to water and sediments (Li et al., 2012; Wu et al., 2021). In eutrophic lakes, the microbial decomposition of a large number of dead aquatic organisms settling to the bottom of the lakes can cause a lower concentration of dissolved oxygen (Wu et al., 2021), which will lead to the production of ammonia, sulfide and other substances (Hu et al., 2023), having a negative impact on the lake ecosystem health (Wang M. et al., 2023; Wang X. et al., 2023).

The N-cycling processes in sediments mainly involve in nitrogen fixation, nitrification, denitrification, assimilatory nitrate reduction (ANRA), dissimilatory nitrate reduction (DNRA) and anammox (Hu et al., 2023), among which nitrification and denitrification are the most important nitrogen transformation processes. These processes induced by microorganisms can oxidize ammonia nitrogen into nitrate nitrogen, and reduce the bound nitrogen into N_2_O or N_2_ back to the atmosphere (Broman et al., 2021). Each pathway of the N-cycling process is completed by the enzyme encoded by the corresponding functional gene using the corresponding substrate catalysis (Broman et al., 2021). However, the abundance and diversity of N-cycling functional genes in lake ecosystems are greatly different due to different water quality (such as water temperature, and nitrogen to phosphorus ratio) (Basu et al., 2022), hydrological conditions (such as lake water exchange cycle) (Stoliker et al., 2016; Li et al., 2021) and seasons (Baumann et al., 2022). Therefore, it is of great significance to explore the changes of N-cycling functional genes in lakes and their influencing factors in different seasons.

Baiyangdian (BYD) Lake (38°43′ ~ 39°02′N, 115°38′ ~ 116°07′E) is the typical eutrophic wetland in North China and has a relatively important geographic position. The BYD Lake water is eutrophicated, accounting for 26.7% of areas “mildly eutrophicated,” accounting for 53.3% of areas “moderately eutrophicated,” and accounting for 20.0% of areas “severely eutrophicated” (Liu et al., 2020; Yao et al., 2023). However, serious eutrophication dominated by seasonal nitrogen and phosphorus pollution occurred due to intense agricultural activities and rural domestic sewage discharge in BYD Lake (Zhao et al., 2011; Cai et al., 2021). Because of the strong exchange between water and surface sediments in shallow lakes, eutrophication might affect the nitrogen cycle in sediments (Shi et al., 2022). The primary objectives of this work were: (1) the key functional genes related to N-cycling have seasonal variability in sediments in the BYD Lake; and (2) some environmental factors can play a key role in regulating the N-cycling process.

## 2 Seasonal variation of N-cycling functional genes

In the current study, a total of 36 sediment samples were collected in four seasons such as spring, summer, fall and winter during 2020–2021 (Supplementary Figure S1). We aimed to identify the major N-cycling gene families and their key environmental factors. A shotgun metagenome sequencing approach was applied to survey 6 important N-cycling processes and related functional genes (Supplementary Text S1): 1) nitrogen fixation (e.g., *nifH, nifD, nifK, vnfG,* and *vnfH*) (Jiang et al., 2022; Li et al., 2022); 2) nitrification (e.g., *amoA, amoB, amoC,* and *hao*) (Wang et al., 2022; Liao et al., 2023); 3) denitrification (e.g., *nirB, nirS, norC, narI and nirK,* and *nosZ*) (Waldrop et al., 2023); 4) DNRA (e.g., *napA, napB, narG, narH, narI, nrfH, nrtA, nirB, nirD, and nrfA*) (Jiang et al., 2023; Waldrop et al., 2023); 5) ANRA (e.g., *nasA, nasD, nirA,* and *nasE*) (Hu et al., 2023; Li et al., 2023); and 6) anammox (e.g., *nirK* and *nirS*) (Tu et al., 2017; Wang M. et al., 2023; Wang X. et al., 2023).

**Figure 1:**
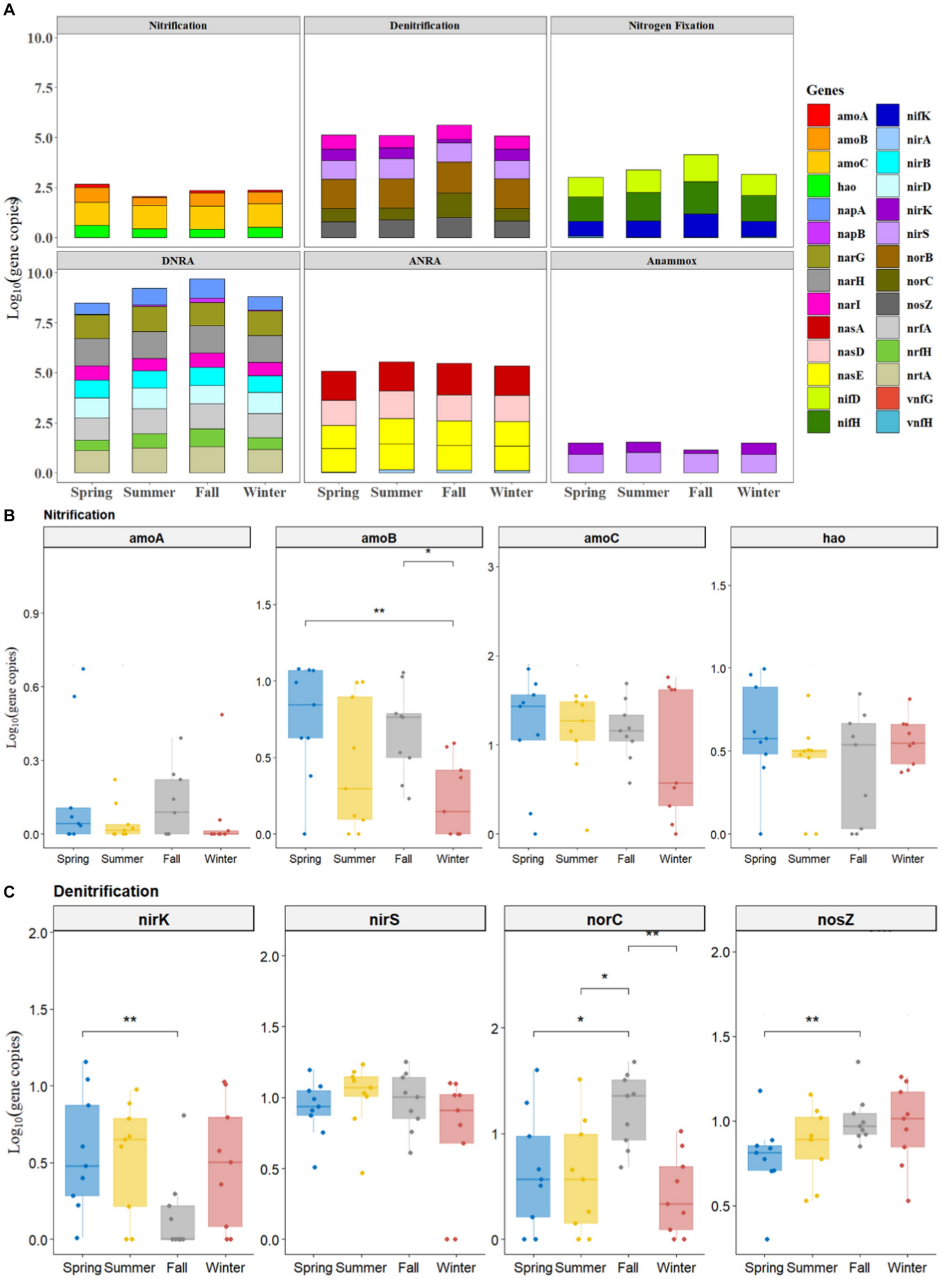
Seasonal profiles of all functional genes of nitrogen metabolism with 6 pathways **(A)** and nitrification genes **(B)** and denitrification genes **(C)**.

All the studied genes of N-cycling (including nitrification, denitrification, nitrogen fixation, DNRA, ANRA, and anammox) were present in the sediments of BYD Lake, although their abundance varied largely among four sampling seasons (Figure 1 and Supplementary Figures S2–S5). According to the results, the functional genes abundance of each N-cycling process followed the order DNRA > ANRA > denitrification > nitrogen fixation > nitrification > anammox (Figure 1A). In general, the abundances of functional genes involved in DNRA, ANRA and denitrification processes were higher than those of other related N-cycling processes, indicating that the sediments in BYD Lake had higher potential of NDRA, ADRA and denitrification. Interestingly, the functional genes of these three N-cycle processes exhibited higher abundance in fall than in other seasons (Figure 1A).

Overall, Figures 1B,C illustrated the seasonal variation of nitrification genes (e.g., *amoA, amoB, amoC, and hao*) and denitrification genes (e.g., *nirk, nirS, norC,* and *nosZ*) processes. Among the nitrification and denitrification genes, the abundance of *nirS* (from 0.47 to 1.96 log_10_ gene copies) and *amoC* (from 0.14 to 1.95 log_10_ gene copies) exceeded the abundance of other functional genes by at least one order of magnitude (Figures 1B,C). Meanwhile, the abundances of such functional genes as *nirS, nirK* and *amoC* genes generally showed a consistent decreasing trend from spring, summer, and fall to winter, while, the abundances of *nosZ* demonstrated an increasing trend, which ranged from 0.31 to 1.68 log_10_ gene copies.

A significantly higher abundance of nitrification gene (*amoB*) in spring (1.083 ± 0.10 log_10_ gene copies, *p* < 0.01) and fall (0.746 ± 0.19 log_10_ gene copies, *p* < 0.05) samples were observed than those in winter (0.42 ± 0.12 log_10_ gene copies) (Figure 1B). As for denitrification, the abundance of *nirK* had significantly higher values in spring (0.63 ± 0.43 log_10_ gene copies) than that in fall (0.37 ± 0.28 log_10_ gene copies) (*p* < 0.01, Figure 1C). On the contrary, the abundances of denitrification genes such as *nosZ* (1.15 ± 0.35 log_10_ gene copies, *p* < 0.01) and *norC* (1.32 ± 0.38 log_10_ gene copies, *p* < 0.05) in fall were significantly higher than those in spring (Figure 1C).

Moreover, significantly higher abundance of nitrogen fixation functional genes (e.g., *nifD, nifH*, and *nifK*) (Supplementary Figure S2), DNRA gene (*napB*, Supplementary Figure S3), ANRA gene (*nasA*, Supplementary Figure S4) in fall were observed than those observed in spring (*p* < 0.05). However, no significant differences were observed in functional genes involved in anammox among spring, summer, fall and winter (*p* > 0.05, Supplementary Figure S5).

## 3 Environmental factors influencing N-cycling functional genes in sediments

**Figure 2:**
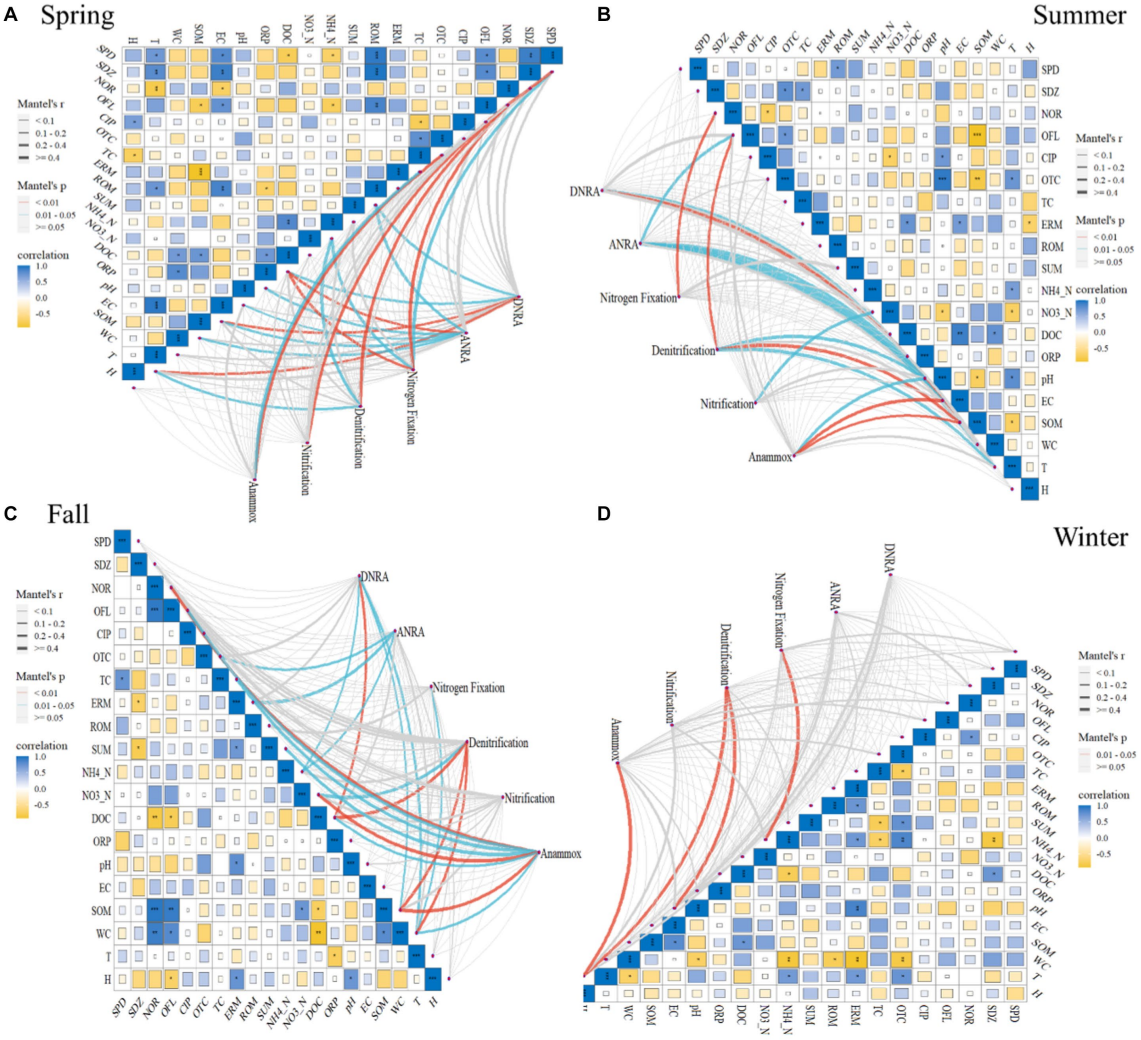
Relationships between functional genes and selected environmental factors, as revealed by the Mantel test in the Spring **(A)**, Summer **(B)**, Fall **(C)** and Winter **(D)**. The edge width is proportional to Mantel’s p value, and the edge color indicates statistical significance. Pairwise correlations of edaphic variables were evaluated by Pearson’s correlation and visualized by a heatmap with a color gradient (correlation coefficients from -1 to 1 correspond to colors from yellow to navy blue, respectively).

The relationships between selected environmental factors and abundances of studied N-cycling functional genes in sediments of BYD lakes are illustrated in Figure 2. In spring, the denitrification pathway was highly correlated with norfloxacin (NOR), NH_4__N and T (r ≥ 4, *p* = 0.01–0.05, Figure 2). In contrast, the nitrification pathway had a significant correlation with sulfapyridine (SPD) (r ≥ 4, *p <* 0.01, Figure 2). Pearson correlation analysis results showed that both denitrification functional genes *nirS* (r = −0.7) and *nosZ* (r = 0.7) were significantly correlated with tetracycline (TC) and oxytetracycline (OTC) (*p <* 0.05, Supplementary Figure S6), while both *nirS* (r = −0.8) and *norB* (r = 0.9) were significantly correlated with pH (*p <* 0.05). Generally, the *norC* abundance exhibited a significant correlation with antibiotics (NOR, r = −0.8; Ofloxacin, OFL, r = 0.7; roxithromycin, ROM, r = 0.7) (*p <* 0.05, Supplementary Figure S6) and some physical–chemical properties (NH_4__N, r = −0.7; DOC, r = −0.8; EC, r = 0.8; SOM, r = −0.7; T, r = 0.8) (*p <* 0.05, Supplementary Figure S6). There were statistically significant positive correlations between nitrification gene *hao* and antibiotics (SDZ, r = 1; OFL, r = 0.7, ROM, r = 0.9) (*p <* 0.05, Supplementary Figure S6), as well as EC (r = 0.8, *p <* 0.05). Besides, the nitrification gene *amoB* was significantly correlated with the pH (r = 0.8, *p <* 0.05, Supplementary Figure S6).

In summer, the denitrification pathway was significantly correlated with OFL (r ≥ 4, *p <* 0.01, Figure 2), pH (r ≥ 4, *p* = 0.01–0.05, Figure 2), SOM (r ≥ 4, *p* = 0.01–0.05, Figure 2), T (r ≥ 4, *p* = 0.01–0.05, Figure 2), respectively. In contrast, the nitrification pathway only showed significant correlations with NO_3__N (r ≥ 4, *p* = 0.01–0.05, Figure 2). Furthermore, the correlation analysis results also showed that OFL, SOM, pH, and T have significant correlations with such denitrification genes as *nirK, norC and narl* (*p <* 0.05, Supplementary Figure S6).

In fall, the Mantel test results showed that denitrification pathway was correlated with NO_3__N (r ≥ 4, *p* = 0.01–0.05, Figure 2), DOC (r ≥ 4, *p* < 0.01, Figure 2), SOM (r ≥ 4, *p <* 0.01, Figure 2), and WC (r ≥ 4, *p <* 0.01, Figure 2), respectively. WC had a significant correlation with such denitrification genes as *nirS* (r = −0.7), *norC* (r = −0.9), *narl* (r = 0.7), and *nosZ* (r = −0.8) and nitrification gene *hao* (r = −0.7) (*p <* 0.05, Supplementary Figure S6). Additionally, DOC (r = 0.7) and SOM (r = −0.9) exhibited a significant correlation with *norC* and *hao* (*p <* 0.05, Supplementary Figure S6). Significant correlations were observed between denitrification gene *nirS* and NOR (r = −0.9, *p <* 0.01), OFL (r = −0.8, *p <* 0.01), TC (r = −0.7, *p <* 0.05), total antibiotics (SUM) (r = −0.8, *p <* 0.05). In contrast, in winter, the denitrification pathway was significantly correlated with EC and H (r ≥ 4, *p* = 0.01–0.05, Figure 2). The denitrification gene *norC* and EC (r = −0.7, *p <* 0.05), *nosZ* and NO_3__N (r = 0.7, *p <* 0.05), nitrification gene *amoC* and CIP (r = −0.7, *p <* 0.05) showed a significant correlation (Supplementary Figure S6). Overall, no significant correlation was observed between the nitrification pathway and environmental factors in fall and winter (*p* > 0.05, Figure 2). What’s more, the DNRA, ANRA, and anammox pathways were correlated with DOC or SOM (*p <* 0.05), and the nitrogen fixation pathway was correlated with NOR (*p <* 0.05).

## 4 Discussion

It is well known that many N-cycling processes are mediated by N-related microorganisms (Isobe and Ohte, 2014). The nitrification and denitrification functional gene abundance could be an indicator of nitrification and denitrification activities, which has been demonstrated by previous studies reporting a positive correlation between them (Jiang et al., 2022). Moreover, the synergistic effect is manifested in a positive correlation of their gene abundance because nitrification can provide sufficient nitrate for denitrification (Jiang et al., 2023). In the current study, a strong correlation was also observed between the abundance of functional genes associated with denitrification (e.g., *nirK, nirS,* and *norB*) and nitrification (e.g., *amoA, amoB* and *hao*) pathway in spring, fall and winter (*p <* 0.05). In summer, the supply of nitrate is limited due to higher temperatures and excessive consumption of oxygen by algae and aquatic plants (Zhou et al., 2021), which may be the reason why we did not observe the correlation between functional genes related to nitrification and denitrification processes in summer.

The different responses of the N cycling process to external stresses might be driven by the remodeling of the microbial community, which could be strongly affected by changes in physical–chemical properties (Tan et al., 2022). A previous study has shown that denitrification and DNRA rates were mainly regulated by the abundance of their functional genes (e.g., *nirS, nirK* and *nrfA*), followed by environmental factors (e.g., sediment organic carbon) (Jiang et al., 2023). Marshall et al. (2021) also reported a decrease in anammox functional gene abundance in conjunction with the decreasing organic carbon content. Similarly, in the current study, we also found that most of the N-cycling pathways (including denitrification, DNRA, ANRA, and anammox) were significantly correlated with SOM or DOC due to the influence of plant growth and litter in BYD Lake, the contents of SOM and DOC in sediment are higher in summer and fallMoreover, N-cycling functional genes including *norC, nirK, narI,* and hao showed a significant correlation with DOC (*p <* 0.05). This could be explained by the fact that organic carbon input can stimulate microbial N-cycling as organic carbon acts as an electron donor for various N-reduction pathways in organotrophic N-reducing reactions, such as denitrification (Baumann et al., 2022). Previous studies have also reported that higher available carbon (DOC) can promote denitrification (Stewart et al., 2013; Morse et al., 2014) due to N-cycling microorganisms can utilize organic carbon for mixed nutrient growth (Jiang et al., 2023). This further illustrates that the denitrification process in the BYD sediment is the dominant process.

Furthermore, our result showed that T (°C) significantly correlated with the denitrification pathway (r ≥ 4, *p* = 0.01–0.05, Figure 2) in summer and fall. This also confirms that the nitrogen cycle process is a microbial-dominated process and is therefore more sensitive to temperature. Specifically, a significant correlation was observed between T and *nirK* (r = −0.7), *norC* (r = 0.9), *narl* (r = −0.7) (*p* < 0.05, Supplementary Figure S6). Studies have shown that the abundance of genes related to ANRN and denitrification pathways decreases with increasing temperature (Yang et al., 2023). Pajares et al. (2017) also found that *nirK* is negatively related to T, furthermore, the elevated temperature will increase denitrification rates (Dai et al., 2020). This highlights the importance of temperature as one of the main factors influencing the functional genes related to N-cycling in lakes (Yuan et al., 2023). Therefore, the impact of seasonal changes on N-cycling triggering the retention and emission of nitrogen in the lake should be paid more attention by the management department.

Previous studies have also reported that antibiotic pollution could alter the N-cycling process (Wu J. et al., 2022). For example, sulfadiazine inhibits functional genes related to denitrification and anaerobic ammonium oxidation in sediments (Wang M. et al., 2023; Wang X. et al., 2023). As well as, nitrifier-denitrification rates were inhibited by sulfamethoxazole (Chen et al., 2022). Remarkably, in the current study, N-cycling pathways significantly correlated with antibiotics. For instance, denitrification exhibited a significant correlation with NOR in spring (r ≥ 4, *p* = 0.01–0.05, Figure 2), and OFL in summer (r ≥ 4, *p* = 0.01–0.05, Figure 2). Nitrification was significantly correlated with SPD (r ≥ 4, *p* = 0.01–0.05, Figure 2). Anammox had a significant correlation with TC in spring and fall, and NOR in fall (r ≥ 4, *p* = 0.01–0.05, Figure 2). Our previous study (Zhang et al., 2023) found that NOR and OFL was the main antibiotics in BYD lake sediments, indicating that more attention should be paid to the effect of antibiotics on the N-cycling in the future. Consequently, more concerns should be given to antibiotics pollution in N-cycling studies in eutrophic water bodies.

Given this perspective, DOC or SOM, T and antibiotics (e.g., norfloxacin and ofloxacin) were significantly correlated with N-cycling processes and they might be potentially key factors influencing the seasonal N-cycling processes in lake ecosystems.

## Data availability statement

The original contributions presented in the study are included in the article/Supplementary material, further inquiries can be directed to the corresponding author.

## Author contributions

LZ: Investigation, Visualization, Writing – original draft, Writing – review & editing. JB: Conceptualization, Data curation, Funding acquisition, Methodology, Writing – review & editing. YZ: Writing – review & editing. KZ: Writing – review & editing. YW: Writing – review & editing. RT: Writing – review & editing. RX: Writing – review & editing. MJ: Writing – review & editing.
