# Supplementary material for: Seasonal changes in N-cycling functional genes in sediments and their influencing factors in a typical eutrophic shallow lake, China
Source: Front Microbiol. 2024 Feb 5;15:1363775. doi: 10.3389/fmicb.2024.1363775 (PMC10876089; doi:10.3389/fmicb.2024.1363775)
Supplement: Supplementary file 1 [file Data_Sheet_1.doc]

# Supplementary Material

**Seasonal changes in N-cycling functional genes in sediments and their influencing factors in a typical eutrophic lake shallow lake, China**

Ling Zhang^ab^, Junhong Bai*^a^, Yujia Zhai^a^, Kegang Zhang^c^, Yaqi Wang^a^, Rong Xiao^d^, Milko A. Jorquera^e^

*a School of Environment, Beijing Normal University, Beijing 100875, China*

*b School of Chemistry and Chemical Engineering, Qinghai Normal University, Xining 810008, China*

*c Department of Environmental Engineering and Science, North China Electric Power University, Baoding, China*

*d College of Environment & Safety Engineering, FuZhou University, Fuzhou, China*

*e Laboratorio de Ecología Microbiana Aplicada (EMALAB), Departamento de Ciencias Químicas y Recursos Naturales, Universidad de La Frontera, Temuco, Chile*

# **Text. S1 Materials and methods**

## **1.1 Sample collection and analysis**

To investigate the seasonal changes of functional gene related to N-cycling and key environmental factors in sediments in Baiyangdian (BYD) lake，we carried out sampling campaigns in October 2020 (Fig. S1). Sediment samples were collected from 9 representative sampling sites in spring, summer, fall and winter. Sediment samples were collected by stainless steel static gravity dredger and stored in three parts to determine physicochemical properties, extract DNA for bioinformatics analysis. All the samples were stored in the freezer and brought back to the laboratory as soon as possible. In the laboratory, one part of the sediments was freeze-dried in a vacuum freeze dryer, ground in an agate mortar, passed through a 0.50 μm sieve and sealed in a plastic bag at - 4 °C before obtaining physicochemical properties. the remaining sediment samples were stored at −80°C to extract DNA.

Physicochemical parameters of the sediment samples including pH, EC, water temperature (T), water content (WC), dissolved organic carbon (DOC), sediment organic matter (SOM), particle size, dissolved oxygen (DO, only for water), nitrate-nitrogen (NO_3_^−^-N), ammonium-nitrogen (NH_4_^+^-N) were analyzed. These parameters were determined according to the standard methods recommended in our previous research (Lu et al., 2018; Zhang et al., 2023)

Nine target antibiotics were selected including norfloxacin (NOR), ofloxacin (OFL), ciprofloxacin (CIP), oxytetracycline (OTC), tetracycline (TC), sulfapyridine (SPD), sulfapyridine (SDZ), erythromycin (ERM) and roxithromycin (ROM). The detail of nine antibiotics and extraction according to our previous research (Zhang et al., 2023). Briefly, the antibiotics were analyzed by high-performance liquid chromatography (HPLC) mass-spectrometry (MS) (HPLC–MS/MS). An API 4500 QTrap liquid chromatography-mass spectrometer (Applied Biosystems, Foster City, CA, USA) and Waters BEH–C18 column (2.1 mm × 100 mm, particle size 1.7 μm) were used for the determination. The internal standard method was used to quantify antibiotic concentrations. Procedure blanks and solvent blank were set up in each batch of experiments and parallel samples were set up for extraction and analysis to avoid accidental errors. A detailed information can be seen in Zhang et al. (2022).

## **1.2 DNA extraction and Illumina sequencing**

DNA was extracted according to the method reported by Zhang et al. (2022), with some modifications. In brief, around 0.5 g sediment samples was subjected to DNA extraction using FastDNA SPIN Kit for Soil (MP Biomedicals, Solon, OH, USA) following the instruction manual. The quality of DNA was checked by spectrophotometric analysis using NanoDrop 2000 UV-vis spectrophotometer (Thermo Scientific, Wilmington, USA). The DNA was stored at −20 °C until use.

Polymerase chain reaction (PCR) amplification was performed using the universal primers 338F (ACTCCTACGGGAGGCAGCAG) and 806R (GGACTACHVGGGTWTCTAAT) designed against the V3-V4 region of bacterial 16S rRNA gene in an ABI GeneAmp® 9700 PCR thermocycler (ABI, CA, USA). Amplicon sequencing was performed using the Illumina MiSeq PE300 platform at the Shanghai Majorbio Bio-pharm Technology Co., Ltd, in China. The PCR amplification of 16S rRNA gene was performed, initial denaturation at 95 °C for 3 mins, followed by 30 cycles of denaturing at 95 °C for 30 s, annealing at 55 °C for 30 s and extension at 72 °C for 45 s, and single extension at 72 °C for 10 min. The details of DNA amplification, Illumina sequencing, and raw sequences processing can be seen in Zhang et al.(2022). Amplicon sequence variants (ASVs) were obtained and representative sequence and abundance information for bioinformatics analysis. Alpha diversities were used in our study to evaluate bacterial community richness (i.e., Sobs), diversity (Shannon), evenness (Shannoneven), and Phylogenetic diversity (PD). The sequence data were submitted to NCBI Sequence Read Archive with the accession number PRJNA929306.

## 1.3 Statistical analysis

One-way ANOVA analysis was performed to test the differences in N-cycling functional genes between four seasons. The significance level was described as *p* < 0.05, *p* < 0.01, or *p* < 0.001. Spearman’s correlation was conducted to identify the correlations between the N-cycling functional genes and environmental factors. Statistical analysis was performed using SPSS 24.0 for Windows or in R (v4.1.1, <https://www.r-project.org/>).

# **Figures**


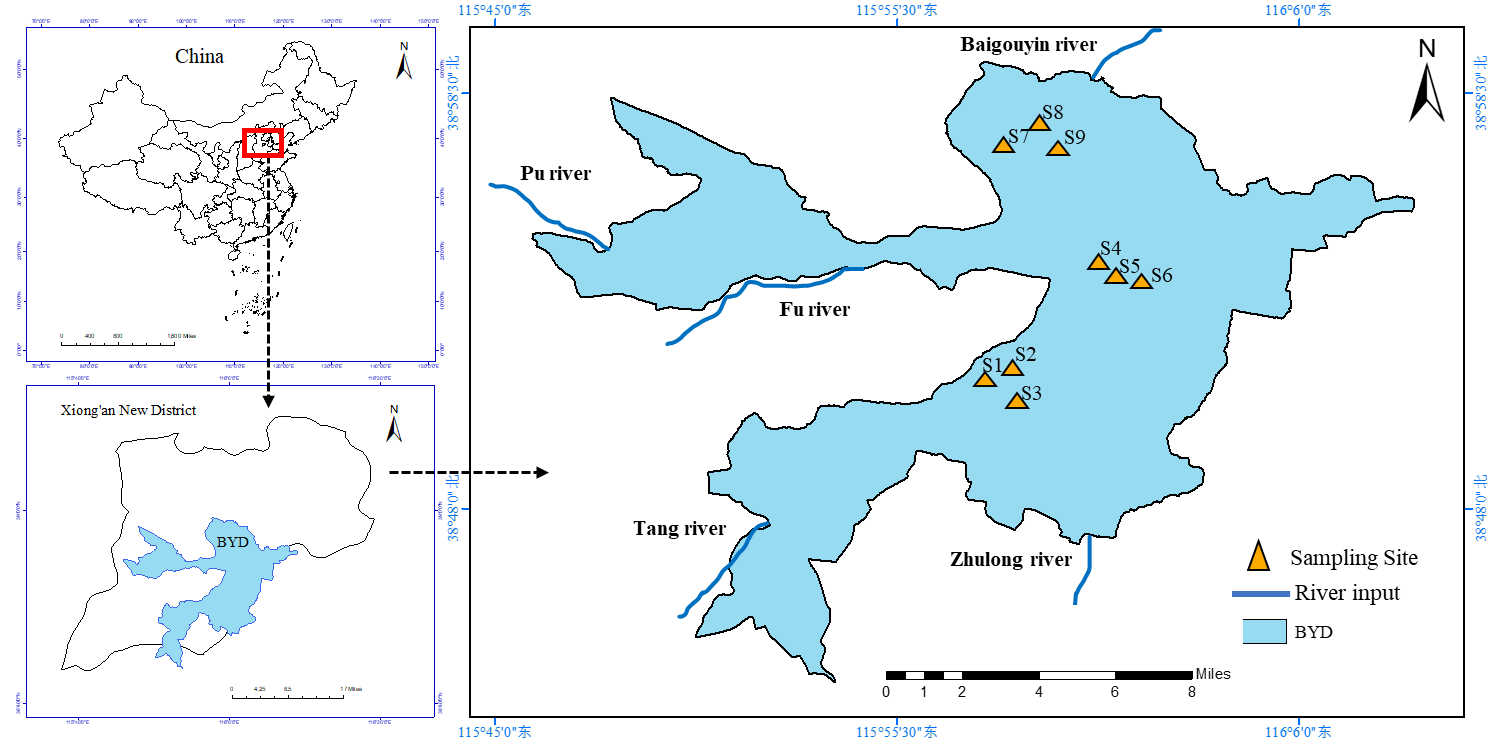


### Fig. S1 Location map of sampling sites in BYD Lake


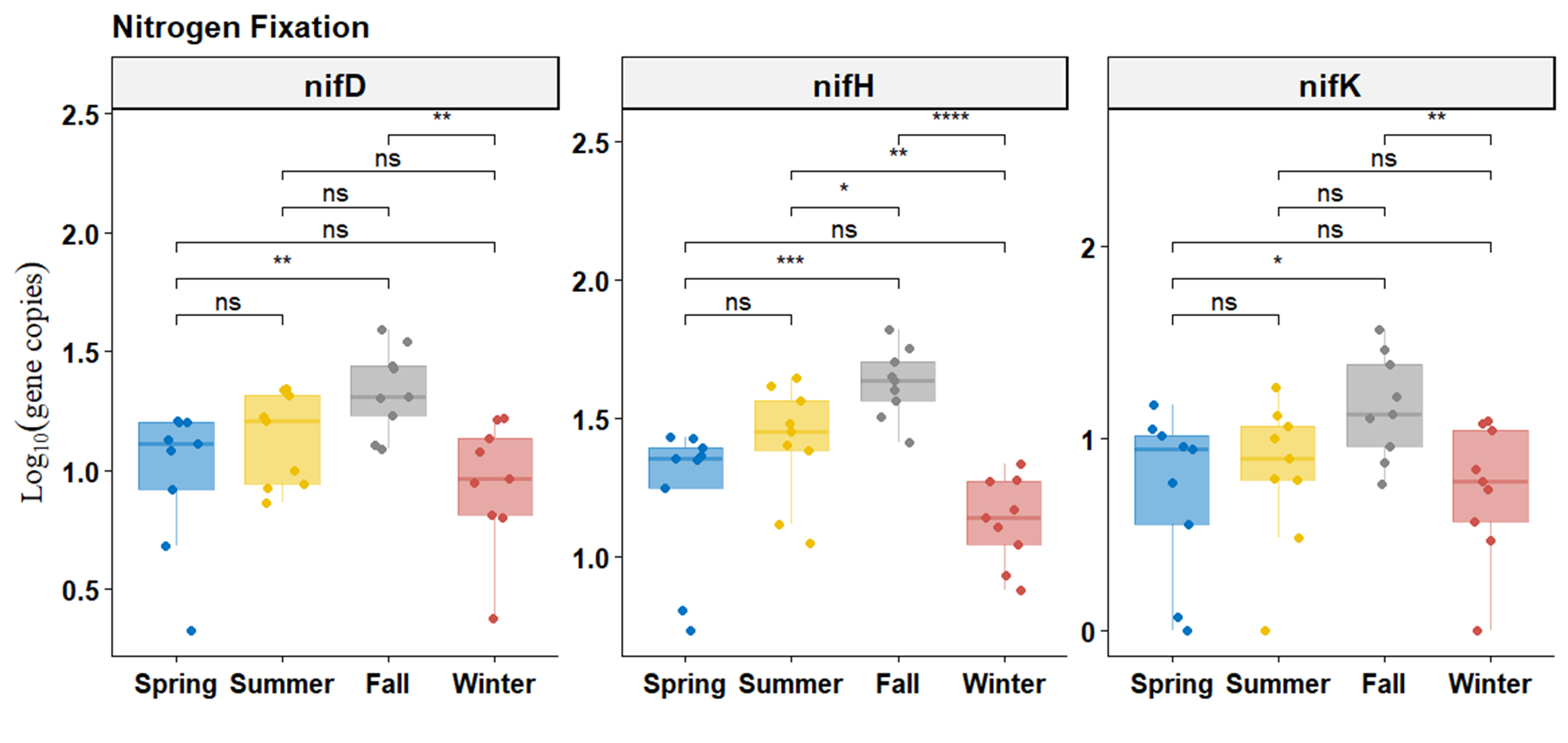


### Fig. S2 Seasonal variation of functional genes related nitrogen fixation process


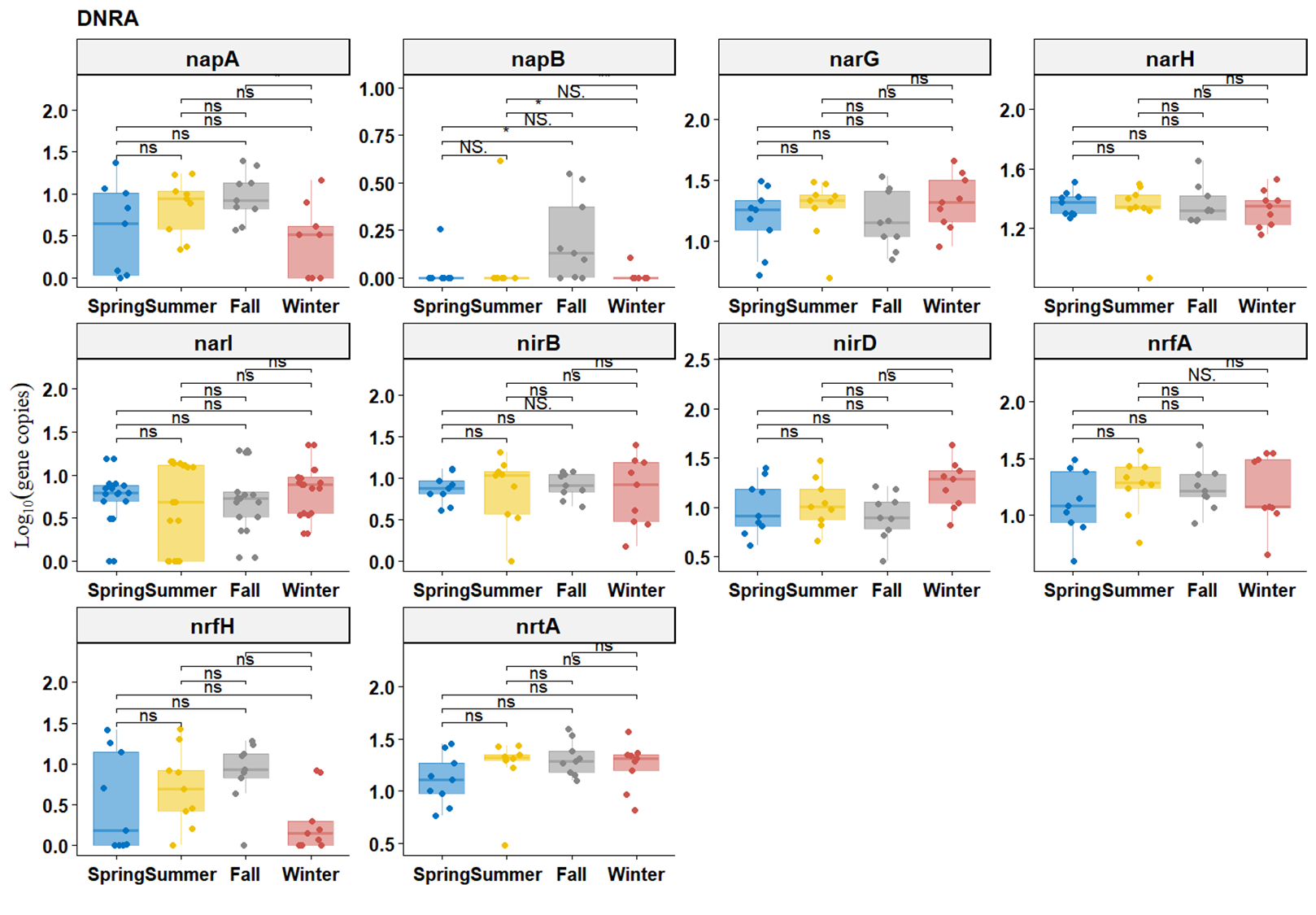


### Fig. S3. Seasonal variation of functional genes related dissimilatory nitrate reduction (DNRA) process


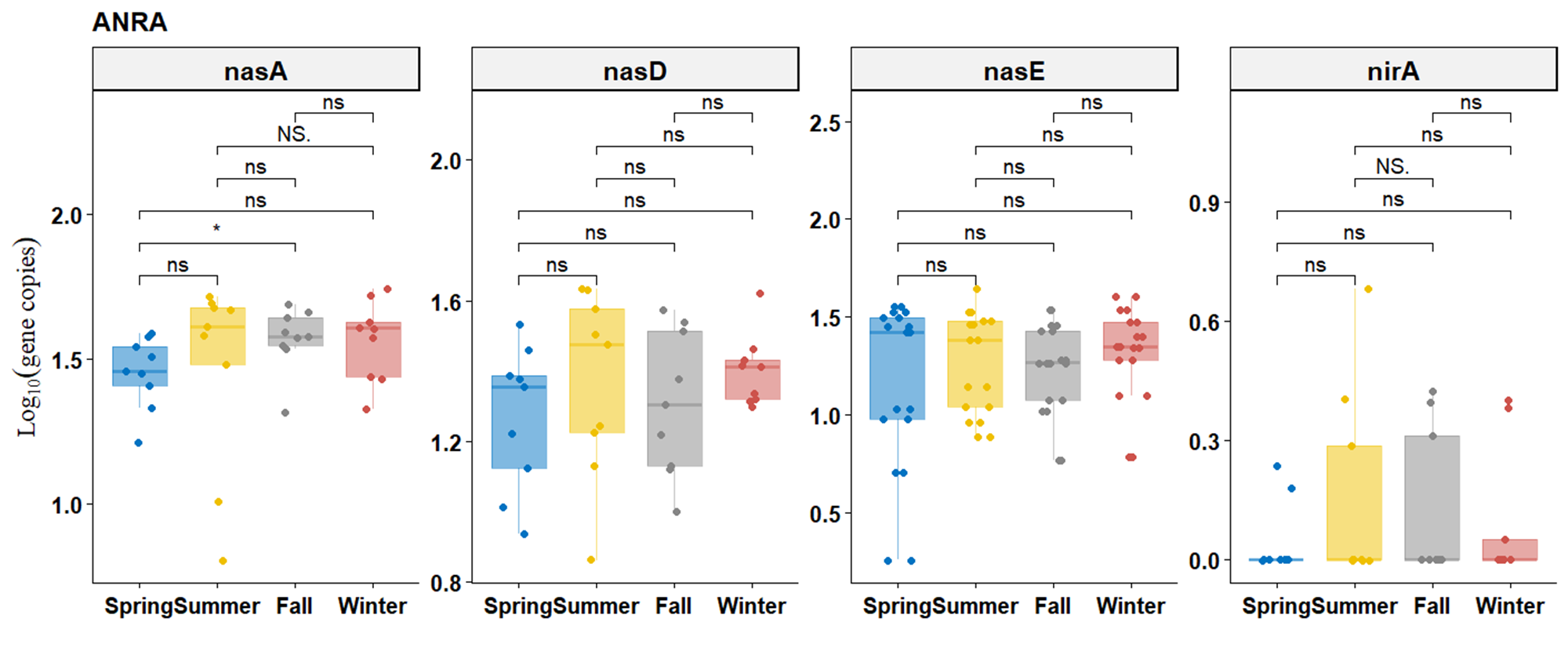


### Fig. S4. Seasonal variation of functional genes related assimilatory nitrate reduction (ANRA) process


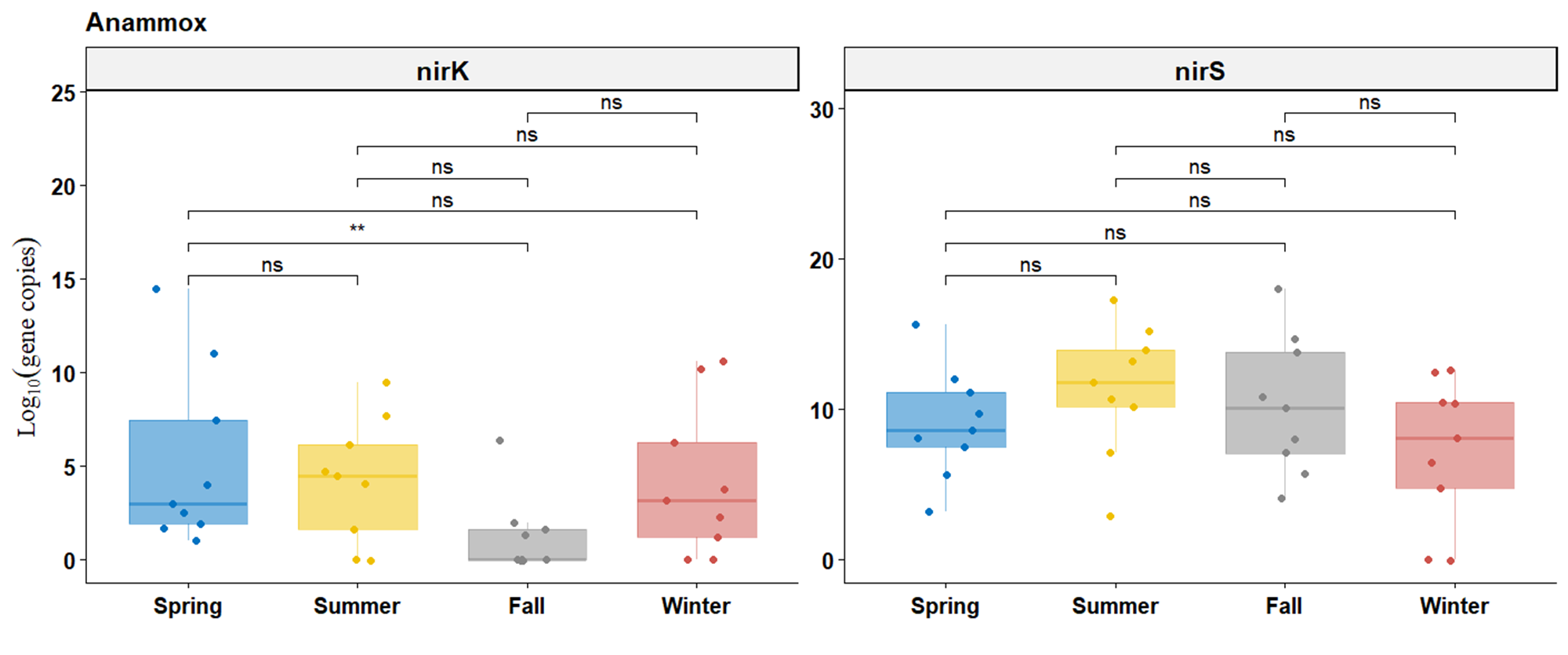


### Fig. S5. Seasonal variation of functional genes related anammox process


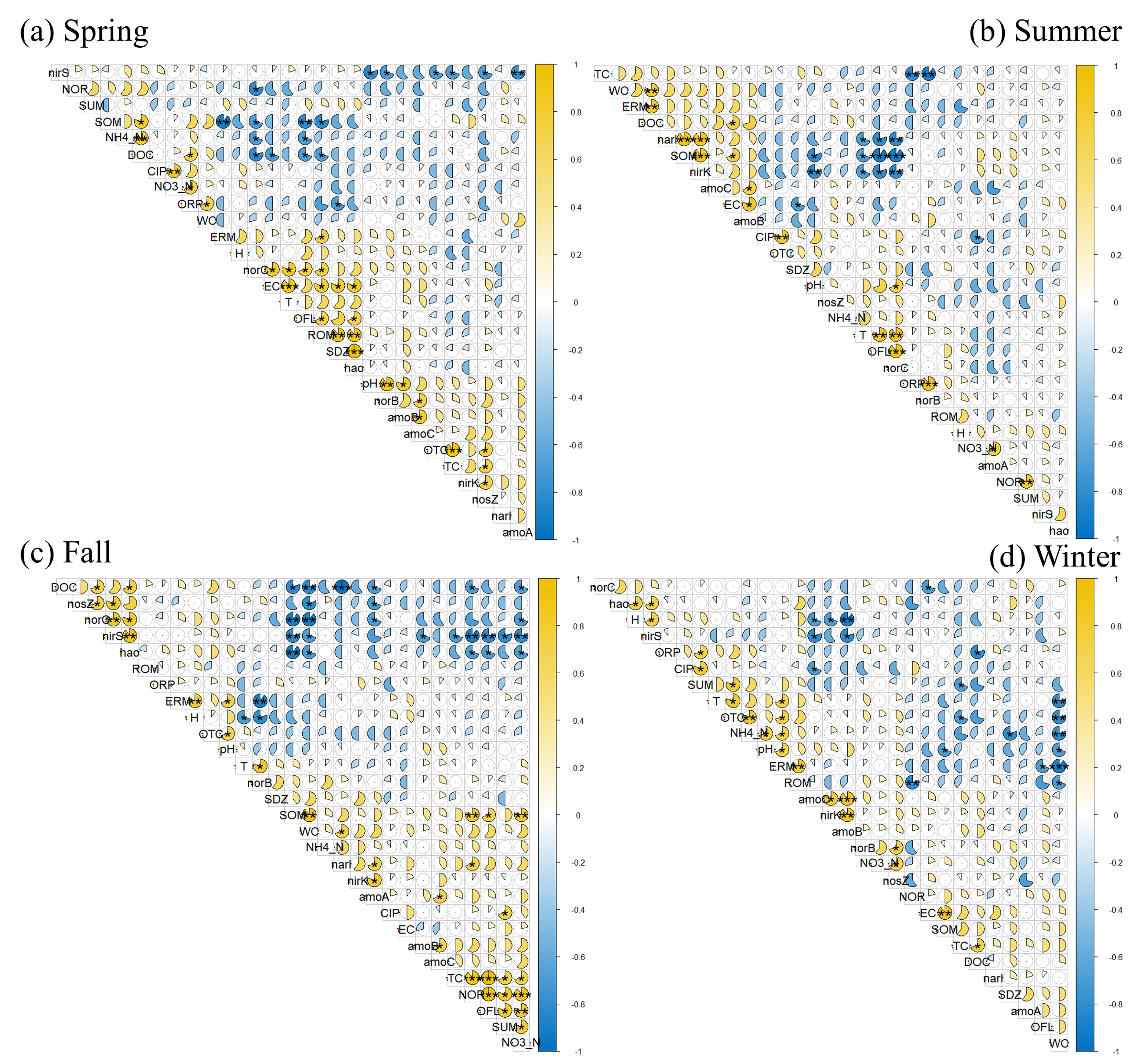


### Fig. S6. Relationships between environmental factors and N-cycling functional genes
